# Effect of solar proton events on test mass for gravitational wave detection in the 24th solar cycle

**DOI:** 10.1038/s41598-023-37005-3

**Published:** 2023-06-19

**Authors:** Ruilong Han, Minghui Cai, Tao Yang, Liangliang Xu, Qing Xia, Xinyu Jia, Dawei Gao, Jianwei Han

**Affiliations:** 1 State Key Laboratory of Space Weather, National Space Science Center, Chinese Academy of Sciences, Beijing, 100190 China; 2 College of Astronomy and Space Science, University of Chinese Academy of Sciences, Beijing, 100049 China

**Keywords:** General relativity and gravity, Space physics

## Abstract

Free-falling cubic Test Masses (TMs) are a key component of the interferometer used for low-frequency gravitational wave (GW) detection in space. However, exposure to energetic particles in the environment can lead to electrostatic charging of the TM, resulting in additional electrostatic and Lorentz forces that can impact GW detection sensitivity. To evaluate this effect, the high-energy proton data set of the Geostationary Operational Environmental Satellite (GOES) program was used to analyze TM charging due to Solar Proton Events (SPEs) in the 24th solar cycle. Using the Geant4 Monte Carlo toolkit, the TM charging process is simulated in a space environment for SPEs falling into three ranges of proton flux: (1) greater than 10 pfu and less than 100 pfu, (2) greater than 100 pfu and less than 1000 pfu, and (3) greater than 1000 pfu. It is found that SPEs charging can reach the threshold within 535 s to 18.6 h, considering a reasonable discharge threshold of LISA and Taiji. We demonstrate that while there is a somewhat linear correlation between the net charging rate of the TM and the integrated flux of $\ge$ 10 MeV SPEs, there are many cases in which the integrated flux is significantly different from the charging rate. Therefore, we investigate the difference between the integral flux and the charging rate of SPEs using the charging efficiency assessment method. Our results indicate that the energy spectrum structure of SPEs is the most important factor influencing the charging rate. Lastly, we evaluate the charging probability of SPEs in the 24th solar cycle and find that the frequency and charging risk of SPEs are highest in the 3rd, 4th, 5th, 6th, and 7th years, which can serve as a reference for future GW detection spacecraft.

## Introduction

Gravitational waves (GWs) are a revolutionary prediction of Einstein’s theory of general relativity. By detecting and researching GWs, the mysteries of dark matter and dark energy can be uncovered, and potential new physics can be explored. It took humankind a century from the publishing of the theory of general relativity to detect GWs directly, which was achieved by the advanced Laser Interferometric Gravitational-Wave Observatory (LIGO) in 2015^1, 2^. Since then, LIGO has detected dozens of GW events from binary black holes and binary neutron star mergers, yet such ground-based detections are limited to GW signals > 10 Hz due to noise effects^3–5^. It has been found that low and intermediate frequency bands (0.1 mHz–1 Hz) contain a range of celestial wave sources since the late 1990s^6–9^, prompting several countries to develop space laser interferometric gravitational waves detection programs, such as LISA^10^, Taiji^11^, TianQin^12^, ASTROD-GW^13^, and DECIGO^14^.

Ground-based and space-based laser interferometric GW detectors are similar in terms of measurement principles, but the latter must guarantee the drag-free motion of the test mass (TM) and achieve the detection of weak tidal strain in spacetime with a long baseline in space. In order to meet its intended targets, the associated noise sources of space GW detection must be controlled, including TM charging noise, thruster disturbances, internal temperature disturbances, and residual gas effects around the TM. The space environment is a critical factor causing GW measurement noise in space, and simulations have been used to estimate the effects of space plasma^15^, galactic cosmic rays^16–18^, and solar cosmic rays^19^. Space plasma effects can be suppressed by optical methods, and Galactic and solar particles with energies above 100 MeV/n can penetrate the spacecraft shield and affect the TM directly. To minimize the intensity of the Coulombic and magnetic forces acting on the TM with higher charge deposition, periodic discharge of the spacecraft in orbit is achieved by ultraviolet light beam irradiation of the TM^20, 21^.

Solar Proton Events (SPEs) are powerful forces of nature with the potential to charge a TM to levels beyond the tolerance of gravitational wave detection. Despite this connection, the exact role of solar flares in SPEs and the mechanism of proton acceleration remain under debate^22, 23^. The LISA program, a collaboration between NASA and the European Space Agency, is composed of three identical spacecraft orbiting the Sun at a distance of 2.5 million kilometers, following Earth by approximately 20$^{\circ }$. Similarly, China’s Taiji program also consists of three spacecraft orbiting in heliocentric orbit at a distance of 3 million kilometers, leading Earth by about 20$^{\circ }$^24^. Monte Carlo simulations on Geant4 and Fluka have been used to evaluate the impact of several typical SPEs on LISA, with the charge rate potentially reaching tens of thousands^25, 26^. This charge accumulation can reach the tolerance limit of gravitational wave detection within minutes. Two upcoming space-based GW observatories-LISA and Taiji-are scheduled for launch in 2034 and 2033, respectively, and are expected to operate for approximately four years, coinciding with the peak of solar activity^27^. The LISA TMs exhibit a time-dependent change in position while in orbit, with their distance from the sun ranging from approximately 149 to 152 $\times$ 10$^6$ km (0.9933–1.0133 AU), and their latitude varying by approximately 1$^{\circ }$ relative to the ecliptic plane^28^. The Ulysses mission has successfully conducted an extensive investigation of the interplanetary medium and solar wind with respect to heliographic latitude. In particular, Ulysses has identified a gradient of roughly 3% per astronomical unit and (0.33 ± 0.04)% per degree for galactic protons with energies surpassing 106 MeV, detected in both the northern and southern hemispheres. Based on these findings, it may be inferred that the anticipated variations between cosmic-ray observations obtained in close proximity to the earth and the LISA spacecraft are negligible when contrasted with typical experimental outcomes. Therefore, data acquired at the earth may be utilized to evaluate the charging mechanism of the LISA TMs^28, 29^. At present, the Geostationary Operational Environmental Satellite (GOES) program, a joint effort between NOAA and NASA, is the main source of solar particle observations and monitors near-Earth space for operational meteorology and space weather^30^. We simulated the charging process of all 41 SPEs of the 24th solar cycle using the Geant4 toolkit based on the High Energy Proton and Alpha Detector (HEPAD) data in GOES. We evaluated the charging rates at different phases of the 24th solar cycle in order to calculate the charging hazard of SPEs at various periods during solar activity. We provide the charging time required to reach the discharge threshold for LISA and Taiji TMs. Furthermore, we address the discrepancy between the integrated flux and charging rate of $\ge$ 10 MeV SPEs by utilizing the charging efficiency assessment method proposed by Ara$\acute{\textrm{u}}$jo et al. with the charging efficiency curve of the proton energy spectrum^25^. We evaluate the distribution and charging risk of SPEs during different periods of solar activity in the 24th solar cycle.

## GOES data

The Geostationary Operational Environmental Satellites (GOES) series is a United States weather satellite system. The satellites’ data is available online by way of NOAA’s National Centers for Environmental Information Satellite Data Service^31^. During Solar Cycle 24, GOES 11 (launched in May 2000), GOES 13 (launched in May 2006) and GOES 15 (launched in March 2010) operated as GOES-West (135$^{\circ }$W), GOES-East (75$^{\circ }$W) and GOES-West (135$^{\circ }$W), respectively. GOES 13 is the primary source of SPEs data, with GOES 11 and GOES 15 being used to supplement GOES 13’s measurements. GOES 13 has six energy channels, two alpha channels, and four proton channels; all of which are composed of High-Energy Proton and Alpha Detectors (HEPADs). From December 2008 until December 2019, HEPADs’ proton differential energy channel data was recorded, which covers the entire Solar Cycle 24. The HEPADS of the GOES satellite is comprised of two detectors: one eastward (HEPAD-A) and one westward (HEPAD-B). The difference in the detected fluxes between the two HEPAD detectors during the SPE is attributed to the east-west effect^32^. The proton detector of the GOES satellite was designed to detect numerous particle counts, but did not come with any active shielding for the anticoincidence system, thus permitting interference from side and rear incident particles. R. Zwickl proposed an algorithm to eliminate this interference which considers the energy range of each energy channel and is able to reject contaminating signals^33^. In this paper, the calibrated 5-min averaged HEPAD monthly data is used. The geometrical factor of HEPAD is approximately 0.73 m$^{2}$ sr. The HEPADs consist of four proton energy channels, including the integral channel P11, whose nominal energy range, average energy, geometrical factor and energy resolution are tabulated in Table 1.

**Table 1:** The GOES 11-15 HEPAD channels P8-P11.

Channel	Energy range (MeV)	Mean energy (MeV)	Geometrical factor (cm$^{2}$ sr MeV)	Energy resolution (%)
P8	330–420	375	67.5	24
P9	420–510	465	67.5	19
P10	510–700	605	162	31
P11	> 700	–	1565	–

Since 1976, GOES satellites have been gathering data on solar protons events while in geostationary orbit. On 14 August 2010, GOES 13 was missing proton data which was substituted with GOES 11. Additionally, GOES 13 was missing data on 22 May 2013 and 29 October 2015, and these were supplemented with GOES 15.

## Solar energetic particles

Protons make up 90% of solar energetic particles (SEPs), followed by alpha particles and a few heavy ions. SEPs were not discovered until the 1940s due to the necessity of certain observational instruments^34^. Nowadays, numerous satellites carry high-energy proton detectors, such as the GOES satellite in the US and the FY satellite in China^35, 36^. SEPs have an energy spectrum from a few keV to a few GeV. SEP events are divided into two categories: impulsive and gradual^22^. In general, impulsive SEP events of less than 1 h are related to solar flares and can reach an energy of up to 50 MeV^26^. Gradual SEP events, which last from a few hours to a few days, are associated with coronal mass ejections (CMEs) and can have an accelerated particle energy of up to several GeV^37^. These properties of gradual SEP events can be a major factor in the charging of free-falling test masses in space-based gravitational wave observatories.

To accurately assess the TM charging process caused by SEP events, we use energy spectrum fitting of GOES satellite high-energy proton detector data. We define an SPE in which the flux of protons with energy greater than 10 MeV exceeds 10 pfu for more than 15 min as observed in Earth Geostationary orbit. SPEs are linked to sunspots, solar flares, and CMEs. The simulation needs to give the peak flux of the SEP energy spectrum. We focus on the energy spectrum at the arrival of the excitation wave, where particles are hypothesized to be generated by a first-order Fermi acceleration mechanism. This process is essentially random and multiplicative, as particles gain energy by scattering freely between the converging upstream and downstream plasma. The solar proton spectrum generated by the stretched exponential (SE) function model (also known as the Weibull like shape) can be represented by the following equation^38^:

1 $$\begin{aligned} \frac{{\mathrm{{d}}J}}{{\mathrm{{d}}E}} = k{E^{(b - 1)}}\exp {( - E/{E_0})^b}. \end{aligned}$$

where *E* is the primary energy, *J* is differential flux, and *k*, $E_0$ and *b* are free parameters.

**Figure 1:**
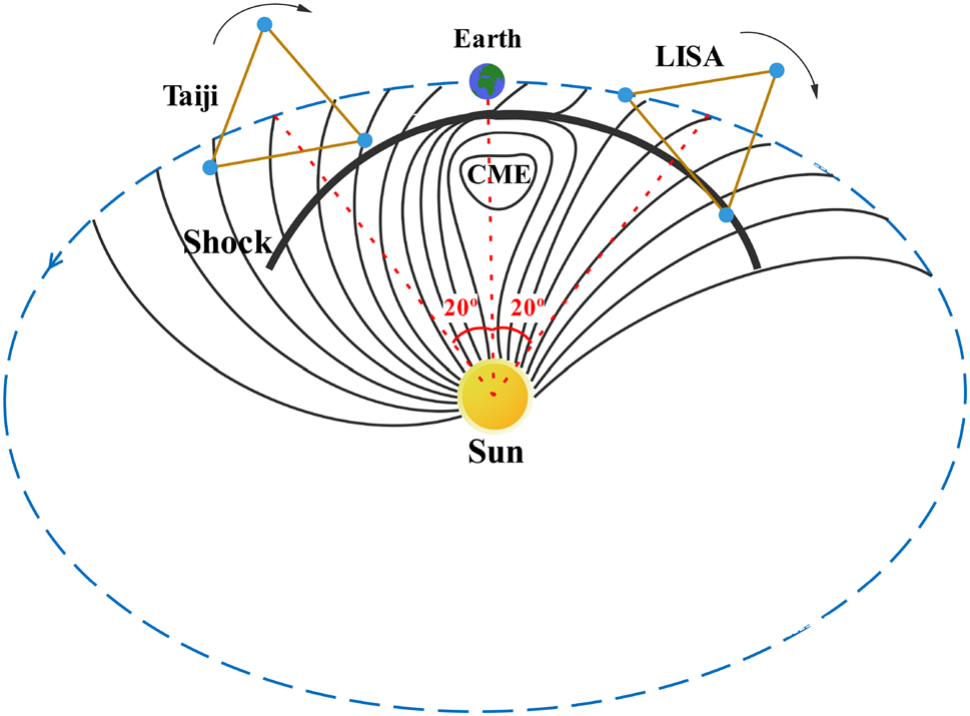
Constructing LISA and Taiji orbits and CME shock propagation.

**Figure 2:**
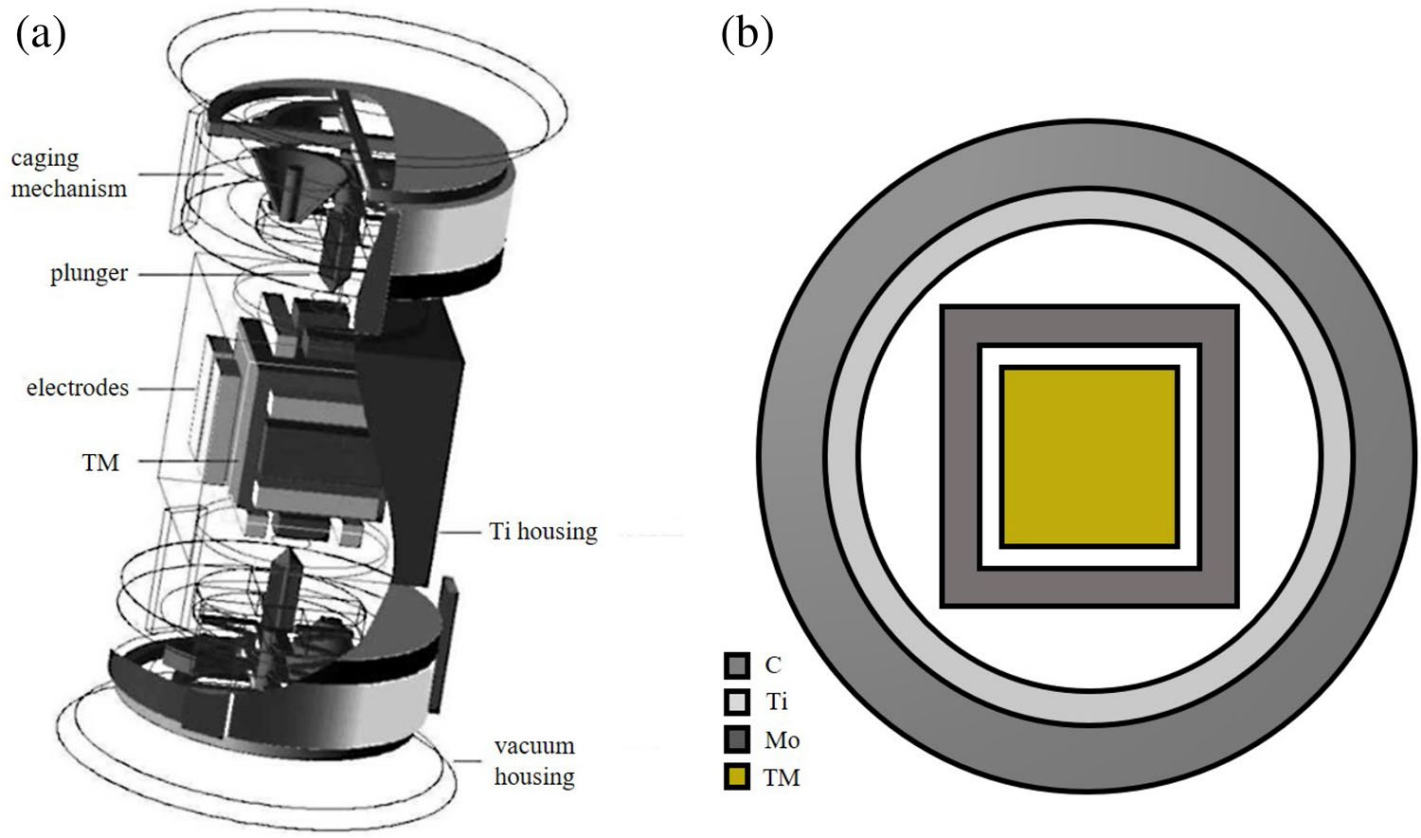
(**a**) LISA inertial sensor model; (**b**) Equivalent simplified model in this paper.

**Table 2:** Spacecraft geometric dimensions and material composition.

Name	Composition	Density (g/cm$^{3}$)	Thickness(mm)
TM	Au (70%); Pt (30%)	19.837	–
Molybdenum electrode	Mo	10.28	6
Titanium housing	Ti	4.54	5
Carbon shell	C	2.1	20

As depicted in Fig. 1, the characteristics of both the LISA and Taiji experiments orbits have been recorded, and the evolution of CME shocks has been demonstrated^39^. Gradual SEP events associated with CMEs have the potential to penetrate the shielding of gravitational wave spacecraft. During such events, the energetic proton flux at the LISA and Taiji orbits can sharply surge by thousands of times the background in a short amount of time.

## Geant4 simulation

### Geometry model

We use a physical model constructed with GEANT4 from previous work^18, 25^. As shown in Fig. 2, we equivalently simplify the complex spacecraft and inertial sensor models based on the GEANT4 inertial sensor model of LISA^25^. The main structural materials, dimensions, and density information of the model are shown in Table 2. The TM is located in the center and is made of a 46 mm $\times$ 46 mm $\times$ 46 mm cube of gold-platinum alloy, with a cube of molybdenum shell layer on the outer layer of the TMs to equate the surrounding molybdenum electrode. The molybdenum shell layer is placed inside a spherical shell layer made of titanium, replacing the high vacuum titanium housing. The carbon layer on the outside is equivalent to other structures of the spacecraft, such as batteries, telescope tubes and mounts. In this paper, the vacuum density is 1.0 $\times$ 10$^{-25}$ g/cm$^{3}$ and the cosmic ray particles are injected uniformly into the entire spacecraft model from a sphere of 120 mm radius.

### SPE radiation environment

**Figure 3:**
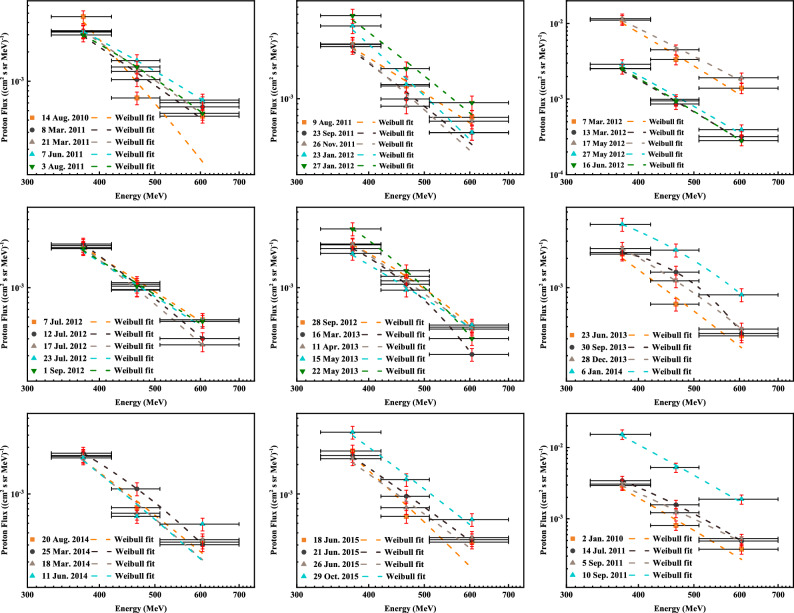
GOES data and energy spectrum for each SPE peak in the solar cycle 24.

SPE fluxes significantly differ at different distance positions from the Sun. The gravitational wave detection spacecraft’s outer shield is sufficient to withstand the impact of 100 MeV protons, and we only consider the charging process of TM with energies greater than 100 MeV protons. Moreover, the gravitational wave spacecraft is in an Earth-like orbit around the Sun, and it is subjected to essentially the same SPE events as high altitude earth orbiting spacecraft^29^. The GOES high-energy proton detector data conform in all aspects to the requirements. We obtain the peak energy spectra of all 41 SPEs in the 24th solar cycle as shown in Fig. 3, using the three high-energy proton detectors P8, P9 and P10 channels of the GOES satellite as the data sources and parametrizing them with the Weibull like shape (also known as the SE function model)^38, 40^. The peak fluxes of the three GOES channels are also plotted in the figure. Figure 3 demonstrates that the Weibull functional form can accurately fit SPEs of varying magnitudes, with a goodness-of-fit index $R^2$ greater than 0.95. We have reported the parameters *k*, $E_0$ and *b* for the parametric GOES satellite data in Table 3. Fitting average error is typically below 20%, although there are five events in which the fitting average error exceeds 20% in Table 3. These discrepancies are largely attributed to the high level of uncertainty in the GOES satellite data.

**Table 3:** Parameterization of SPEs energy spectrum in the solar cycle 24.

Event no.	Date of event	I ($>10$ MeV) (pfu)	*k*	*b*	$E_0$	$R^2$	Fitting average error (%)
1	2010/08/14 12:30	14	8.63 $\times$ 10$^{6}$	3.31 $\times$ 10$^{-1}$	6.51 $\times$ 10$^{-2}$	0.976	40.53
2	2011/03/08 01:05	50	5.63 $\times$ 10$^{5}$	2.20 $\times$ 10$^{-1}$	1.96 $\times$ 10$^{-3}$	0.991	17.83
3	2011/03/21 19:50	14	4.07 $\times$ 10$^{5}$	2.25 $\times$ 10$^{-1}$	3.00 $\times$ 10$^{-3}$	1	11.36
4	2011/06/07 08:20	72	1.73 $\times$ 10$^{1}$	4.70 $\times$ 10$^{-1}$	1.02 $\times$ 10$^{1}$	1	0.10
5	2011/08/04 06:35	96	8.09 $\times$ 10$^{-1}$	7.39 $\times$ 10$^{-1}$	5.64 $\times$ 10$^{1}$	1	0.02
6	2011/08/09 08:45	26	3.87 $\times$ 10$^{3}$	2.58 $\times$ 10$^{-1}$	5.64 $\times$ 10$^{-2}$	0.995	10.44
7	2011/09/23 22:55	35	7.09 $\times$ 10$^{5}$	2.17 $\times$ 10$^{-1}$	1.58 $\times$ 10$^{-3}$	0.993	15.72
8	2011/11/26 11:25	80	3.25 $\times$ 10$^{7}$	1.98 $\times$ 10$^{-1}$	1.56 $\times$ 10$^{-4}$	0.971	28.73
9	2012/01/23 05:30	6310	1.80 $\times$ 10$^{6}$	2.56 $\times$ 10$^{-1}$	8.70 $\times$ 10$^{-3}$	0.997	12.84
10	2012/01/27 19:05	796	1.19 $\times$ 10$^{6}$	2.22 $\times$ 10$^{-1}$	2.10 $\times$ 10$^{-3}$	0.993	15.80
11	2012/03/07 05:10	6530	4.34 $\times$ 10$^{6}$	2.38 $\times$ 10$^{-1}$	3.88 $\times$ 10$^{-3}$	0.996	14.71
12	2012/03/13 18:10	469	2.88 $\times$ 10$^{5}$	2.46 $\times$ 10$^{-1}$	8.02 $\times$ 10$^{-3}$	0.998	8.91
13	2012/05/17 02:10	255	5.91 $\times$ 10$^{5}$	2.22 $\times$ 10$^{-1}$	3.34 $\times$ 10$^{-3}$	0.998	7.67
14	2012/05/27 05:35	14	3.49 $\times$ 10$^{5}$	2.37 $\times$ 10$^{-1}$	5.22 $\times$ 10$^{-3}$	0.997	9.82
15	2012/06/16 19:55	14	3.14 $\times$ 10$^{4}$	2.98 $\times$ 10$^{-1}$	8.42 $\times$ 10$^{-2}$	0.999	1.43
16	2012/07/07 04:00	25	6.22 $\times$ 10$^{4}$	2.25 $\times$ 10$^{-1}$	5.25 $\times$ 10$^{-3}$	0.999	5.26
17	2012/07/12 18:35	96	1.93 $\times$ 10$^{2}$	4.63 $\times$ 10$^{-1}$	4.26 $\times$ 10$^{0}$	1	0.13
18	2012/07/17 17:15	136	4.11 $\times$ 10$^{3}$	3.65 $\times$ 10$^{-1}$	5.92 $\times$ 10$^{-1}$	1	0.11
19	2012/07/23 15:45	12	2.02 $\times$ 10$^{5}$	2.07 $\times$ 10$^{-1}$	1.24 $\times$ 10$^{-3}$	0.995	12.36
20	2012/09/01 13:35	59	8.35 $\times$ 10$^{4}$	2.17 $\times$ 10$^{-1}$	3.02 $\times$ 10$^{-3}$	0.998	6.70
21	2012/09/28 03:00	28	2.39 $\times$ 10$^{-2}$	1.11 $\times$ 10$^{0}$	1.47 $\times$ 10$^{2}$	1	0.06
22	2013/03/16 19:40	16	3.76 $\times$ 10$^{-4}$	1.70 $\times$ 10$^{0}$	2.32 $\times$ 10$^{2}$	1	0.13
23	2013/04/11 10:55	114	2.10 $\times$ 10$^{3}$	3.39 $\times$ 10$^{-1}$	4.71 $\times$ 10$^{-1}$	1	0.17
24	2013/05/15 13:25	41	6.35 $\times$ 10$^{4}$	2.15 $\times$ 10$^{-1}$	2.89 $\times$ 10$^{-3}$	0.998	6.19
25	2013/05/22 14:20	1660	8.13 $\times$ 10$^{-2}$	1.12 $\times$ 10$^{0}$	1.16 $\times$ 10$^{2}$	1	0.01
26	2013/06/23 20:14	14	8.05 $\times$ 10$^{5}$	2.27 $\times$ 10$^{-1}$	2.30 $\times$ 10$^{-3}$	0.993	16.92
27	2013/09/30 05:05	182	4.56 $\times$ 10$^{-7}$	2.62 $\times$ 10$^{0}$	3.65 $\times$ 10$^{2}$	1	0.16
28	2013/12/28 21:50	29	2.73 $\times$ 10$^{-1}$	8.37 $\times$ 10$^{-1}$	7.82 $\times$ 10$^{1}$	1	0.17
29	2014/01/06 09:15	1033	4.53 $\times$ 10$^{-3}$	1.34 $\times$ 10$^{0}$	2.21 $\times$ 10$^{2}$	1	0.02
30	2014/02/20 08:50	22	9.03 $\times$ 10$^{5}$	2.37 $\times$ 10$^{-1}$	3.74 $\times$ 10$^{-3}$	0.994	16.55
31	2014/02/25 13:55	103	6.55 $\times$ 10$^{-2}$	1.03 $\times$ 10$^{0}$	1.14 $\times$ 10$^{2}$	1	0.03
32	2014/04/18 15:25	58	1.89 $\times$ 10$^{6}$	2.44 $\times$ 10$^{-1}$	4.27 $\times$ 10$^{-3}$	0.987	24.57
33	2014/09/11 02:40	126	4.37 $\times$ 10$^{5}$	2.68 $\times$ 10$^{-1}$	1.64 $\times$ 10$^{-2}$	0.959	32.56
34	2015/06/18 11:35	17	4.05 $\times$ 10$^{6}$	2.67 $\times$ 10$^{-1}$	9.47 $\times$ 10$^{-3}$	0.984	29.48
35	2015/06/21 21:35	1070	8.70 $\times$ 10$^{4}$	2.52 $\times$ 10$^{-1}$	1.42 $\times$ 10$^{-2}$	0.999	4.62
36	2015/06/26 02:30	22	6.62 $\times$ 10$^{5}$	2.23 $\times$ 10$^{-1}$	1.97 $\times$ 10$^{-3}$	0.992	17.26
37	2015/10/29 05:50	24	8.66 $\times$ 10$^{5}$	2.40 $\times$ 10$^{-1}$	5.07 $\times$ 10$^{-3}$	0.997	11.05
38	2016/01/02 04:30	22	1.54 $\times$ 10$^{6}$	2.41 $\times$ 10$^{-1}$	4.10 $\times$ 10$^{-3}$	0.992	19.36
39	2017/07/14 09:00	22	2.02 $\times$ 10$^{-1}$	9.05 $\times$ 10$^{-1}$	9.36 $\times$ 10$^{1}$	1	0.05
40	2017/09/05 00:40	844	1.38 $\times$ 10$^{5}$	2.19 $\times$ 10$^{-1}$	2.98 $\times$ 10$^{-3}$	0.998	7.46
41	2017/09/10 16:45	1490	1.42 $\times$ 10$^{6}$	2.52 $\times$ 10$^{-1}$	1.08 $\times$ 10$^{-2}$	0.998	7.89

**Table 4:** Simulation parameters and results of SPEs for solar cycle 24.

Event no.	Date of event	I (> 10 MeV) (pfu)	Exposure time (s)	$$R_{net}$$ (+e/s)	$$R_{eff}$$ (e/s)	$$S_R$$ (e/s/Hz$$^{1/2})$$
1	2010/08/14 12:30	14	11.08	1425 ± 7	1519	55.12
2	2011/03/08 01:05	50	5.82	3025 ± 32	3304	81.29
3	2011/03/21 19:50	14	5.33	3317 ± 23	3687	85.87
4	2011/06/07 08:20	72	14.11	1297 ± 7	1515	55.05
5	2011/08/04 06:35	96	18.19	1046 ± 5	1194	48.88
6	2011/08/09 08:45	26	11.08	1593 ± 20	1858	60.96
7	2011/09/23 22:55	35	5.98	2924 ± 36	3227	80.34
8	2011/11/26 11:25	80	3.54	4781 ± 33	5295	102.91
9	2012/01/23 05:30	6310	2.12	8156 ± 37	8770	132.44
10	2012/01/27 19:05	796	3.02	5797 ± 29	6406	113.19
11	2012/03/07 05:10	6530	1.14	15174 ± 135	16750	183.03
12	2012/03/13 18:10	469	5.82	2963 ± 26	3332	81.63
13	2012/05/17 02:10	255	1.94	9053 ± 76	10220	142.97
14	2012/05/27 05:35	14	5.62	3092 ± 27	3456	83.14
15	2012/06/16 19:55	14	5.63	3131 ± 19	3455	83.12
16	2012/07/07 04:00	25	9.26	1933 ± 11	2150	65.58
17	2012/07/12 18:35	96	7.34	2486 ± 20	2752	74.19
18	2012/07/17 17:15	136	5.46	3278 ± 36	3566	84.45
19	2012/07/23 15:45	12	9.77	1807 ± 11	2037	63.83
20	2012/09/01 13:35	59	10.08	1747 ± 17	2027	63.67
21	2012/09/28 03:00	28	29.94	640 ± 3	754	38.84
22	2013/03/16 19:40	16	47.41	401 ± 3	484	31.13
23	2013/04/11 10:55	114	7.63	2318 ± 15	2653	72.85
24	2013/05/15 13:25	41	11.84	1489 ± 8	1703	58.37
25	2013/05/22 14:20	1660	13.43	1455 ± 9	1697	58.26
26	2013/06/23 20:14	14	6.73	2568 ± 26	2888	75.99
27	2013/09/30 05:05	182	101.50	149 ± 1	201	20.03
28	2013/12/28 21:50	29	23.42	805 ± 7	940	43.35
29	2014/01/06 09:15	1033	26.40	679 ± 5	836	40.90
30	2014/02/20 08:50	22	5.35	3273 ± 27	3558	84.36
31	2014/02/25 13:55	103	24.46	780 ± 4	913	42.74
32	2014/04/18 15:25	58	4.12	4153 ± 19	4527	95.16
33	2014/09/11 02:40	126	4.32	4008 ± 19	4363	93.42
34	2015/06/18 11:35	17	2.32	7204 ± 81	7724	124.29
35	2015/06/21 21:35	1070	7.12	2450 ± 16	2770	74.43
36	2015/06/26 02:30	22	7.15	2410 ± 19	2693	73.39
37	2015/10/29 05:50	24	3.30	5209 ± 44	5802	107.73
38	2016/01/02 04:30	22	3.93	4359 ± 7	4822	98.20
39	2017/07/14 09:00	22	18.34	1036 ± 7	1201	49.01
40	2017/09/05 00:40	844	7.75	2317 ± 10	2590	71.98
41	2017/09/10 16:45	1490	0.94	18685 ± 163	20425	202.11

### Physics models

The high-energy and hadron properties of SEPs imply that they are incident on spacecraft and undergo complex physical processes with spacecraft materials resulting in a large number of secondary charged particles. By tracking these secondary charged particles to the lowest possible energy, the charging process of the TM is more accurately simulated. In this paper, the Monte Carlo simulations are performed using the GEANT4 toolkit version geant4.10.03.p03. GEANT4 is used to simulate a physical model containing a range of energies from several hundred eV to TeV, and to track each particle and all the secondary particles it generates throughout. The different types and energies of particles are closely related to the corresponding physical processes, which are considered: low-energy electromagnetic processes (G4EmStandardPhysics_option4), hadron processes (G4HadronPhysicsQGSP_BIC, G4IonPhysics and G4HadronElasticPhysics), decay processes (G4DecayPhysics), electro-nuclear and gamma-nuclear processes (G4EmExtraPhysics). As the energy of the particles increases, the physical processes that occur with the spacecraft material also change. All particles produced in the simulation are tracked to zero energy, but to avoid the problem of divergence due to too many multilevel particle production. GEANT4 sets the minimum threshold for the production of secondary particles (e−, e+, gamma) from low-energy electromagnetic processes to 250 eV (corresponding to a cutoff length of about 50 nm). For hadron ionization, the cutoff energy is usually the average mean ionization energy of the material, e.g., 790 eV for gold. however, in order to refine the particle-matter interaction process as much as possible, we use 250 eV as the cutoff energy for e−, e+, gamma.

**Figure 4:**
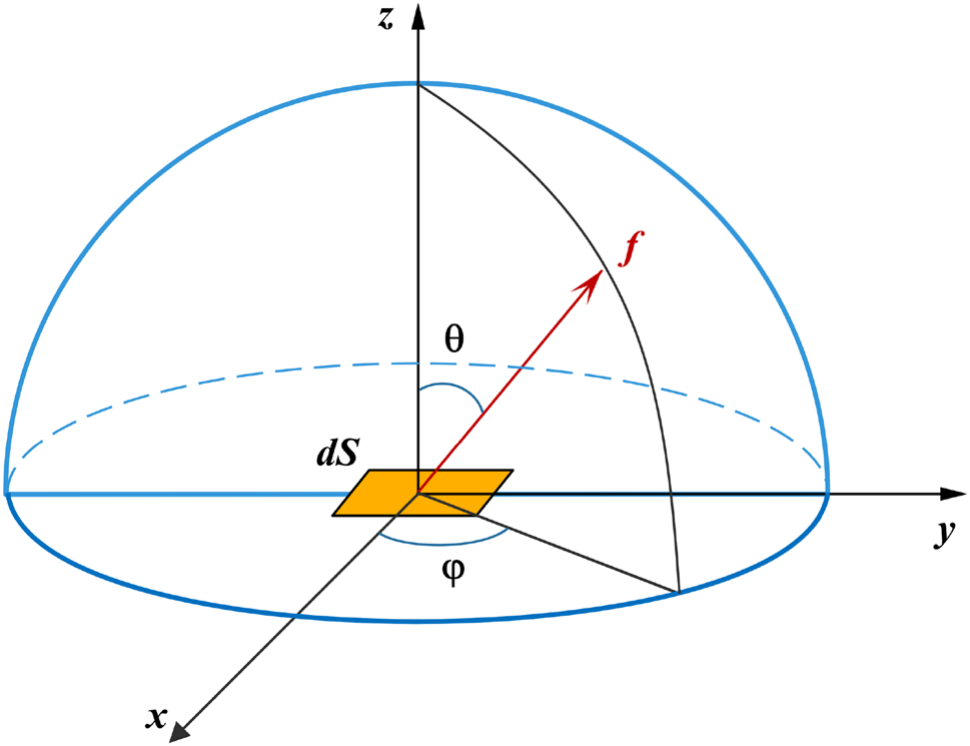
Schematic diagram of the surface source *dS* emitting flux *f* particles.

**Figure 5:**
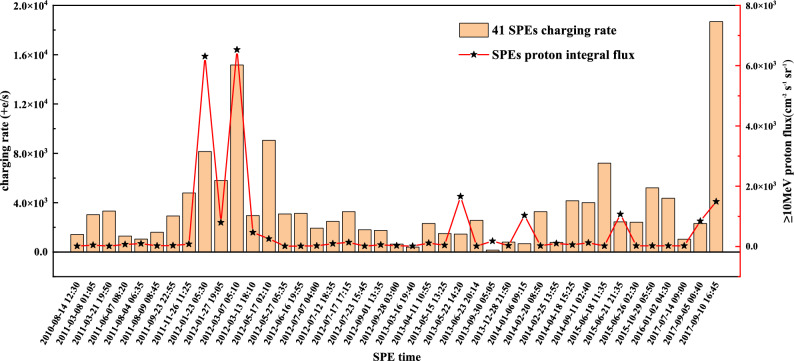
SPEs charging rate and flux of $\ge$ 10 MeV protons in solar cycle 24.

**Figure 6:**
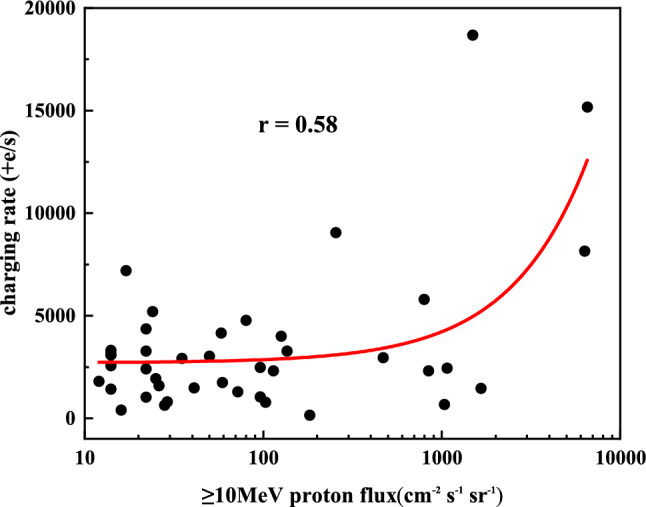
Correlation of SPEs charging rate and $\ge$ 10 MeV proton flux.

**Figure 7:**
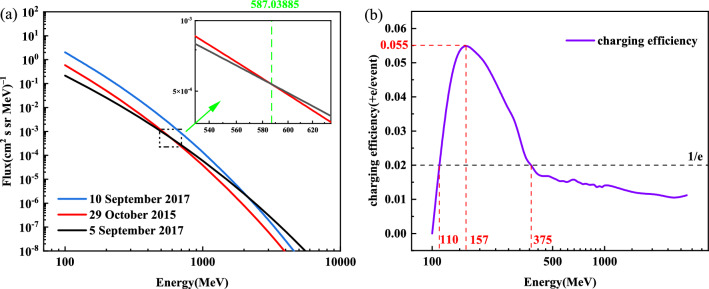
(**a**) Energy spectrum of three typical SPEs; (**b**) proton charging efficiency with energy.

**Figure 8:**
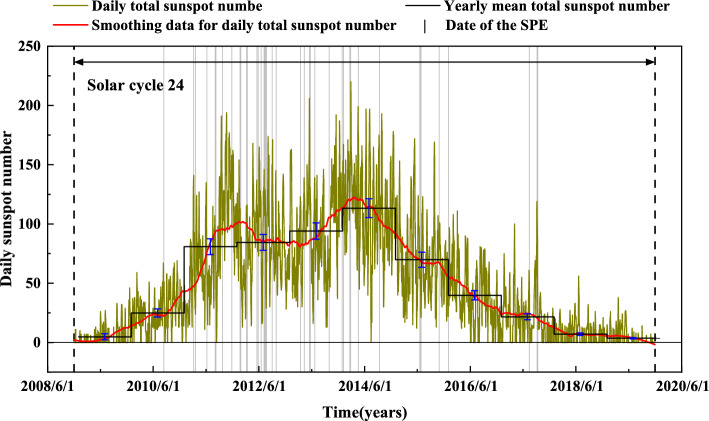
Sunspot number data and SPEs data in solar cycle 24.

**Figure 9:**
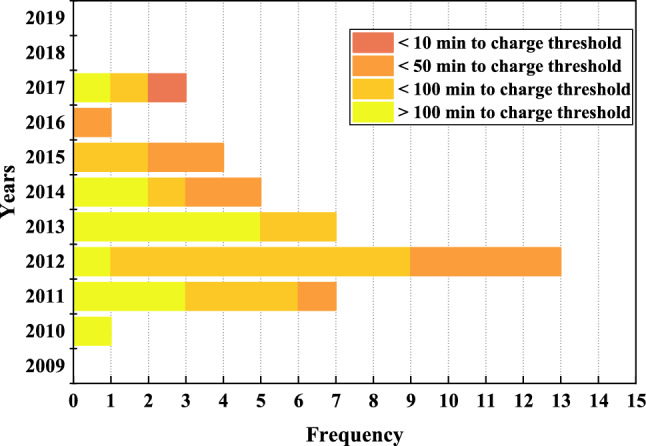
SPEs intensity distribution in solar cycle 24.

## Simulation results and discussion

The LISA simplified geometry model used in this paper was originally presented in previous work^18^. We simulated all SPEs in solar cycle 24 with an energy range of 100 MeV–30 GeV. The simulation input requires energies greater than 100 MeV/n to penetrate the external shielding of the TM. SPE spectra sharply decay above 30 GeV and their flux is too low to make a significant contribution to TM charging. The SPE data utilized in this paper is sourced from the GOES satellite, providing an early warning platform for solar activity. This paper adopts the GEANT4 simulation tool to consider Li to Ca, proton, $^{3}$He, $^{4}$He, e−, e+, deuterium, tritium and $\pm \pi$ mesons in the statistical TM charge, allowing for a thorough analysis of the TM’s charging. Monte Carlo simulations have been shown to be accurate in previous studies^41, 42^, although there is an error margin of ±30% due to imprecision of the solar proton energy spectrum model, physical processes, and the spacecraft model.

### SPEs charging

We have conducted an examination of the average net charging rate ($R_{net}$) and effective charging rate ($R_{eff}$) of the TM in the LISA simplified model as affected by SPEs. Specifically, we define the net charging rate deposited in the TM using the following expression:

2 $$\begin{aligned} R_{n e t}=\sum _{j=-\infty }^{+\infty } j \lambda _j \ \mathrm {~s}^{-1}. \end{aligned}$$

where *j* represents the net number of positive and negative charges deposited by single events, and $\lambda _j$ is the rate of occurrence of these events. As previously mentioned, in the net charging computation, positive and negative charges cancel out, while in the effective charging, both positive and negative net deposited charges contribute. The effective charging rate is defined by the following equation:

3 $$\begin{aligned} R_{e f f}=\sum _{j=-\infty }^{+\infty } j^2 \lambda _j \ \mathrm {~s}^{-1}. \end{aligned}$$

The effective charging rate, denoted as $R_{eff}$, corresponds to the charging of single charges that produces the same observed shot noise. The spectral density of the charging shot noise is then expressed as a function of the effective charging rate.

4 $$\begin{aligned} S_R=\sqrt{2 e^2 R_{e f f}} \quad {e\;s}^{-1} \mathrm {~Hz}^{-1/2}. \end{aligned}$$

where e is the elementary charge.

The details of the geometric models and materials of LISA and Taiji are still not available. To enable an appropriate spacecraft model for TM charging, an equivalent simplified Geant4 model has been proposed based on the materials and geometry already reported by LISA Pathfinder and LISA. In this work, the SPEs of the entire solar cycle 24 were used as the environmental sample, and the energy spectrum data was shown in Fig. 3 and Table 3. Monte Carlo simulation was used to simulate the charging process of the SPEs to the TM. In Table 4, we have reported the exposure time, the integrated flux, the net charging rate $R_{net}$, the effective charging rate $R_{eff}$, and the charging noise $S_R$ for all SPEs of solar cycle 24. Notably, the energy range of the integrated fluxes in Table 4 is from 100 MeV to 30 GeV, and the exposure time is the true duration of exposure in the space environment. During the charging simulation, it was assumed that the particles were emitted from a spherical surface with a radius of 120 mm in an isotropic and uniform manner onto the geometric model. 500,000 particles were simulated in all 41 SPEs simulations. The charging rate is determined by the total time the spacecraft model is exposed to the SPEs, the isotropic spherical source radius, and the integrated flux. This can be calculated from the total number of different SPE simulations and the number of charges accumulated in the TM.

It is assumed that the total number of different SEP events simulated is $N_0$, the spacecraft exposure time is *T*, the radius of the isotropic spherical source is *r*, the integrated flux per unit stereo angle of SPE is *f* (1/cm$^{2}$ s sr), and the total integrated flux of SPE is *F* (1/cm$^{2}$ s). As shown in Fig. 4, the number of particles per unit time and per unit stereo angle emitted by the surface source *dS* is obtained according to the definition of *f*.

5 $$\begin{aligned} dn = f\cos \theta dSd\Omega . \end{aligned}$$

In the simulation, we only consider the particles emitted by the surface source *dS* to the internal spacecraft model, so we obtain the number of particles emitted by the surface source to the internal spacecraft model per unit time as:

6 $$\begin{aligned} \frac{{dN}}{T} = \int _0^{2\pi } {\int _0^{\pi /2} {f\sin \theta \cos \theta d\theta d\varphi dS = \pi f} } dS. \end{aligned}$$

Integrating over the entire sphere, the above equation is:

7 $$\begin{aligned} \frac{{{N_0}}}{T} = \pi f \cdot 4\pi {r^2}. \end{aligned}$$

So the exposure time of the spacecraft is:

8 $$\begin{aligned} T = \frac{{{N_0}}}{{F \cdot \pi {r^2}}}. \end{aligned}$$

Based on the definitions of net charge rate and effective charge rate, we assume that *j* represents the net number of charges deposited in the TM by a single event. There are a total of $n_j$ such single events. Therefore, $\lambda _j$ can be expressed as follows:

9 $$\begin{aligned} {\lambda _j} = \frac{{{n_j}}}{T} = \frac{{{n_j}}}{{\frac{{{N_0}}}{{F \cdot \pi {r^2}}}}} = \frac{{{n_j}}}{{{N_0}}} \cdot F \cdot \pi {r^2}. \end{aligned}$$

By inserting Eq. (9) into Eqs. (2) and (3), the corresponding charging rate can be determined, and the resulting charging noise can be calculated.

Table 4 shows that the charging rate of the TM by 41 SPEs in solar cycle 24 can vary greatly, ranging from 148.948 +e/s on 30 September 2013 for the 27th to 18685.383 +e/s on 10 September 2017 for the 41st. Six of these events had a charging rate of less than 1000 +e/s, twenty-eight had a rate of more than 1000 +e/s but less than 5000 +e/s, and seven had a rate of more than 5000 +e/s. Table 4 presents the net charge rate and its corresponding uncertainty. The uncertainties are mostly below 1% relative to the net charging rate, and all SPEs result in net charging rate uncertainties of less than 2%. We consider a ± 50% error in the charging rate to account for the uncertainty in the SPE energy spectrum, the uncertainty in the geometric model, and the uncertainty in the Monte Carlo simulation. To highlight the net charging rate of SPEs, we compare them to the charging rates of long-standing Galactic cosmic rays reported in a previous study: solar minimal: 39.469 +e/s; solar maximal: 12.531 +e/s^18^. Most SPEs occur during the solar maximum phase, and the Monte Carlo simulations in this paper demonstrate that the charging rate of SPEs to the TM is ten to thousands of times higher than the charging rate of galactic cosmic rays during the solar maximum phase. These findings are consistent with the results of LISA team research studies, which have reported charging rates ranging from tens to tens of thousands for different SPEs^25, 26^.

The charging process of SPEs is a crucial source of noise in gravitational wave detection experiments. Both LISA and Taiji have an overall acceleration noise limit of $3 \times 10^{-15} \mathrm {~m \ s}^{-2} \mathrm {~Hz}^{-1 / 2}$ at 0.1 mHz^43, 44^. The acceleration noise spectral density arising from electrostatic forces during cosmic ray charging at LISA can be divided into two parts^45^. Firstly, any residual $\Delta _x$ multiplied by random charge noise causes electrostatic force noise at 0.1 mHz:

10 $$\begin{aligned} \begin{aligned} S_{F(\delta q)}^{1 / 2}=&\frac{S_q^{1 / 2}}{C_T}\left| \frac{\partial C_X}{\partial x}\right| \Delta _x \approx 0.36 \times 10^{-15} \frac{\textrm{m}}{\mathrm {s^2} \sqrt{\textrm{Hz}}} \times \left( \frac{\Delta _x}{10 \mathrm {~mV}}\right) \left( \frac{R_{\textrm{eff}}}{300 / \textrm{s}}\right) ^{1 / 2}. \end{aligned} \end{aligned}$$

where $C_T$ is the total capacitance of the TM with respect to its surroundings, $\frac{\partial C_X}{\partial x}$ is the capacitance gradient along *x*, $\Delta _x$ is the potential difference due to TM charging, $S_{F(\delta q)}^{1 / 2}$ is the random charge spectrum noise density, $S_q^{1 / 2}$ is the charge Poissonian shot spectral noise density.

Secondly, the fluctuation of residual $\Delta _x$, when multiplied by any non-zero TM charge, results in electrostatic force noise at 0.1 mHz:

11 $$\begin{aligned} \begin{aligned} S_{F\left( \delta \Delta _x\right) }^{1 / 2}=&\frac{q}{C_T}\left| \frac{\partial C_X}{\partial x}\right| S_{\Delta _x}^{1 / 2} \approx 2 \times 10^{-15} \frac{\textrm{m}}{\mathrm {s^2} \sqrt{\textrm{Hz}}} \times \left( \frac{q}{10^7 e}\right) \left( \frac{S_{\Delta _x}^{1 / 2}}{300 \mu \textrm{V} / \textrm{Hz}^{1 / 2}}\right) . \end{aligned} \end{aligned}$$

Here, $S_{F\left( \delta \Delta _x\right) }^{1 / 2}$ is any nonzero TM charge spectrum noise density, $S_{\Delta _x}^{1 / 2}$ is the stray potential difference spectrum noise density, $10^7$ +e is roughly two days of accumulated charge and a reasonable discharge threshold.

Table 4 presents the effective charging rate and net charging rate for all 41 SPEs in cycle 24. According to Eq. (11), the upper limit of charging noise is considered as the reasonable periodic discharge threshold of $10^7$ e/s (LISA and Taijix have the same threshold). Equation (10) provides the effective charging rate required to reach the charging noise $2 \times 10^{-15} \mathrm {~m \ s}^{-2} \mathrm {~Hz}^{-1 / 2}$ at 0.1 mHz, which is calculated as 9259 e/s. SPEs occurring on March 7, 2012, May 17, 2012, and September 10, 2017 exceeded this threshold. Using Eq. (10), we calculated the time required to reach the reasonable periodic discharge threshold based on the net charge rate reported in Table 4. For the 24th solar cycle SPE with the minimum net charge rate on June 23, 2013, we estimated that it would reach the threshold in $\sim$ 18.6 h. For the 24th solar cycle SPE with the maximum net charge rate on September 10, 2017, we estimated that it would reach the threshold in   535 s. It should be noted that timely discharge measures are necessary in such cases^46^.

### Factors affecting SPE charging rate

As shown in Fig. 5, we have demonstrated the charging pattern and characteristics of SPEs during solar cycle 24 in the form of histograms. The charge rates of all 41 SPEs and the corresponding fluxes and trend lines of $\ge$ 10 MeV protons are also presented. It is evident that the TM charge rates vary greatly for two SPEs with close fluxes; a good example is the 6 January 2014 SPE with a flux of 1033 pfu and the 21 June 2015 SPE with a flux of 1070 pfu, which have respective charging rates of 679 +e/s and 2450 +e/s. This implies that the flux of $\ge$ 10 MeV protons is not a major determinant of the charging rate.

An analysis of the correlation between the charge rate and the $\ge$ 10 MeV proton flux was performed to obtain the relationship between the two variables, as presented in Fig. 6. By utilizing Pearson correlation analysis, the correlation coefficient *r* was calculated to be 0.58, which lies in between 0 and 1, suggesting a certain level of positive correlation between proton flux $\ge$ 10 MeV and the charging rate. However, the correlation analysis for r = 5.8 does not explain the anomalies of individual points within the correlation.

In this study, we adopt Ara$\acute{\textrm{u}}$jo et al.’s approach for estimating the expected charging rate of SPEs by multiplying the energy spectrum charging efficiency with the differential energy spectrum of SPEs^25^, to investigate the impact of the energy spectrum structure of SPEs on charging. We address the issue that the flux of protons with energy $\ge$ 10 MeV cannot completely account for the discrepancy in the charging rates of SPEs. Using the same geometric model as in section “Geometry model”, simulation was performed with an isotropic particle number of 200000 per single energy point. We have obtained the charging efficiency curve (the number of individual single-energy protons charged in the TM) for protons in the energy range of 100 MeV–30 GeV through single-energy proton charging simulations, as shown in Fig. 7b. The charging efficiency curve for protons ranging from 100 MeV to 30 GeV is shown in Fig. 7b. The curve indicates that charging occurs only for proton energies greater than 100 MeV, with the highest charging efficiency observed at an energy of 157 MeV. Additionally, the charging efficiency of protons in the energy range of 110–375 MeV (110 MeV and 375 MeV for 1/e of the peak charging efficiency) is significantly higher compared to other energy bands. SRIM calculations indicate that protons with energies below 385 MeV accumulate directly in the TM, and the average charging efficiency for 110–375 MeV is 2.7 times greater than for protons greater than 375 MeV. Here, we have provided evidence for the claim that the energy spectrum structure is the primary factor affecting the charging rate by selecting three typical events. For the 41 SPEs in solar cycle 24, three SEPs events of different intensities on 5 September 2017, 29 October 2015, and 10 September 2017 were studied to analyze the influence of energy spectra on the estimates of TM charging. The proton energy spectra of these three SEPs can be seen in Fig. 7a. The flux of 844 pfu for the event $\ge$ 10 MeV on 5 September 2017 resulted in a charging rate of 2317 +e/s, the flux of 24 pfu for the event $\ge$ 10 MeV on 29 October 2015 resulted in a charging rate of 5209 +e/s, and the flux of 1490 pfu for the event $\ge$ 10 MeV on 10 September 2017 yielded a charge rate of 18685 +e/s. Notably, the energy spectra of the two SEPs on 5 September 2017 and 29 October 2015 intersect at $\sim$ 587 MeV, with the SEP on 29 October 2015 having higher fluxes of energy spectra below 587 MeV and the SEP on 5 September 2017 having higher fluxes of energy spectra above 587 MeV. The findings indicate that the variation in the charging rate between the event on September 5, 2017, with an integral flux of $\ge$ 10 MeV of 844 pfu, and the event on October 29, 2015, with an integral flux of $\ge$ 10 MeV of 24 pfu is attributable to the difference in the flux of the low energy fraction (< 375 MeV). By combining the proton charging efficiency versus energy curve, the relationship between the magnitude of the TM charging rate for each of these three typical SEPs can be determined.

In conclusion, a linear relationship exists between the $\ge$ 10 MeV proton integral fluxes of different SPEs and the charging rate of TM. However, sporadic SPEs with $\ge$ 10 MeV proton integral fluxes cannot be explained by this relationship. This study addresses this issue by exploring the variation of proton charging efficiency with energy using the expected charging rate calculation method for SPEs proposed by Ara$\acute{\textrm{u}}$jo et al.^25^. A key difference between this study and Ara$\acute{\textrm{u}}$jo et al. is that the latter focuses on the calculation of the expected charging rate without a comparative analysis of the charging energy spectra of different SPEs. In contrast, the charging rate of SPEs is directly calculated from the energy spectrum input GENAT4 in this study, and the relationship between the $\ge$ 10 MeV proton integral flux and the charging rate is discussed separately by calculating the energy spectrum charging efficiency. It is concluded that the energy spectrum structure of SPEs is the main factor determining the charging rate.

### Solar cycle 24 SPE distribution

Solar cycle 24 began in December 2008 with a 13-month smoothed sunspot number of 2.2, peaking in April 2014 with a 13-month smoothed number of 81.8, and ending in December 2019 with a 13-month smoothed minimum of 1.8. This cycle spanned 11 full years, and the daily total sunspot number, the smoothing data for it, and the yearly mean total sunspot number are all displayed in Fig. 8^47^. Additionally, the timing of all 41 SPEs within the cycle are included in the same figure. These events are absent for two years at the beginning and two years at the end of solar cycle 24, but occur in eight of the 11 years. 2011, 2012, 2013, 2014, and 2015 have the highest frequency of SPEs, with 36 events taking place in these four years, comprising 88% of all SPEs. The probability model for the occurrence of SPEs is consistent with the model proposed by Birch and Bromage^48^. Notably, the definition of SPEs employed in this study differs from that proposed by Nymmik, Birch and Bromage^48–50^, resulting in some disparities between observed events and predictions. Furthermore, during a particular period spanning 2011 to 2015, the sum of smoothed monthly mean sunspot numbers is significantly higher than in other years, coinciding with a corresponding pattern of SPEs.

We have systematically calculated and discussed the different TM charging rates caused by various SPEs in the section “Factors affecting SPE charging rate”. The years 2011–2015 have the highest probability of SPE occurrence. As discussed in the “SPEs charging” section, a net charge rate greater than $10^7$ +e is the threshold for a reasonable discharge of the TM. We classified the 41 SPEs of the 24th solar cycle based on the time required to reach the threshold charge, which is divided into four categories: (1) charging to the threshold takes more than 100 min, corresponding to a net charge rate less than 1667 +e/s, (2) charging to the threshold takes less than 100 min, corresponding to a net charge rate greater than 1667 +e/s and less than 3333 +e/s, (3) charging to the threshold takes less than 50 min, corresponding to a net charge rate greater than 3333 +e/s and less than 16667 +e/s, and (4) charging to the threshold takes less than 10 min, corresponding to a net charge rate greater than 16667 +e/s. Figure 9 reports the frequency of the above four types of SPEs in different years. It can be found that charging to the threshold takes less than 100 min, including less than 50 min and less than 10 min, for a total of 28 events, 25 of which occurred in the 5-year period from 2011 to 2015. Therefore, we can conclude that not only were there more SPEs in 2011, 2012, 2013, 2014, and 2015, but they also had a greater intensity of charging. LISA and Taiji are projecting launch dates in 2034 and 2033, respectively, within solar cycle 26. Solar cycle 26 is anticipated to start in March 2031 and conclude in February 2041^51^. This means that the expected launch of Taiji and LISA will take place in the third to fourth year of the solar cycle 26’s commencement, which corresponds to the years 2011 and 2012 of solar cycle 24. In consideration of the fact that it takes about one year from launch to arrival at the designated science exploration position, the actual science run time corresponds to 2012 and 2013 in solar cycle 24—a time when solar activity is highly frequent and the number and intensity of SPEs are at their peak. During this period, the issue of charging of SPEs on TM cannot be disregarded.

## Conclusions

When the gravitational wave spacecraft is in orbit, SPEs have been found to contribute to the charging of the TM, resulting in acceleration noise that affects the precision of scientific detection data. To simulate the charging process of the gravitational wave detection spacecraft for all SPEs energies in the solar cycle 24 (100 MeV–30 GeV) for the TM, a Monte Carlo simulation was performed using high-quality data from GOES high-energy proton detectors. A simplified approximation of the known geometry material surrounding the TM was used, as detailed descriptions of both the Taiji spacecrafts’ models are not currently available. The SPEs data from GOES satellites were fitted to an Weibull function model, which has been proven to be consistent with observations from a previous study. It was found that SPEs charging can reach the threshold within 535 s to 18.6 h, considering the charge tolerance boundaries of LISA and Taiji. Furthermore, we observed a positive correlation (r = 0.58) between the charging rate obtained from simulations and the proton flux of $\ge$ 10 MeV, which helped to evaluate the relationship between SPEs and charging rate. However, during the fitting process, we identified two SPEs with essentially the same flux that led to significantly different charging rates. To better explain these differences, we utilized the method proposed by Ara$\acute{\textrm{u}}$jo et al., which assesses the charging rate based on the charging efficiency of single-energy protons. It was found that the charging efficiency of protons in the energy range 110–375 MeV is significantly higher than the rest of the range. By combining the SPEs energy spectrum and the single-energy proton charging efficiency curves, this discrepancy can be explained. Additionally, we evaluated the frequency and intensity distributions of SPEs in different years of solar cycle 24 and found that the frequency and intensity of SPEs peaked in years 3rd, 4th, 5th, 6th and 7th, which greatly increased the charging risk. With the predicted launch of Lisa and Taiji in years 3rd and 4th of solar cycle 26, and the arrival at the assigned position in years 4th and 5th of solar cycle 26, the charge of SPEs must be taken into consideration.

## Supplementary Information

Supplementary Information.

## Data Availability

High-energy solar proton data obtained through the NOAA’s National Centers for Environmental Information Satellite Data (https://www.ngdc.noaa.gov/stp/satellite/goes/dataaccess.html, accessed on 21 November 2022). All simulation input and output data is available in supplementary information.

## References

[1] Weber J (1960). Detection and generation of gravitational waves. Phys. Rev..

[2] Abbott BP (2016). Observation of gravitational waves from a binary black hole merger. Phys. Rev. Lett..

[3] Abbott B (2019). GWTC-1: A gravitational-wave transient catalog of compact binary mergers observed by LIGO and Virgo during the first and second observing runs. Phys. Rev. X.

[4] Abbott R (2021). GWTC-2: Compact binary coalescences observed by LIGO and Virgo during the first half of the third observing run. Phys. Rev. X.

[5] Abbott, R. *et al.* GWTC-3: Compact binary coalescences observed by LIGO and Virgo during the second part of the third observing run. arXiv preprint arXiv:2111.03606 (2021).

[6] Schutz BF (1999). Gravitational wave astronomy. Class. Quantum Gravity.

[7] Miller MC (2002). Gravitational radiation from intermediate-mass black holes. Astrophys. J..

[8] Berti E, Cardoso V, Will CM (2006). Gravitational-wave spectroscopy of massive black holes with the space interferometer LISA. Phys. Rev. D.

[9] Sathyaprakash BS, Schutz BF (2009). Physics, astrophysics and cosmology with gravitational waves. Living Rev. Relativ..

[10] Amaro-Seoane, P. *et al.* Laser interferometer space antenna. arXiv preprint arXiv:1702.00786 (2017).

[11] Gong X (2015). Descope of the ALIA mission. J. Phys. Conf. Ser..

[12] Luo J (2016). TianQin: A space-borne gravitational wave detector. Class. Quantum Gravity.

[13] Ni W-T (2013). ASTROD-GW: Overview and progress. Int. J. Mod. Phys. D.

[14] Kawamura S (2011). The Japanese space gravitational wave antenna: DECIGO. Class. Quantum Gravity.

[15] Su W (2021). Analyses of laser propagation noises for TianQin gravitational wave observatory based on the global magnetosphere MHD simulations. Astrophys. J..

[16] Grimani C (2005). Lisa test-mass charging process due to cosmic-ray nuclei and electrons. Class. Quantum Gravity.

[17] Villani M, Benella S, Fabi M, Grimani C (2020). Low-energy electron emission at the separation of gold-platinum surfaces induced by galactic cosmic rays on board LISA pathfinder. Appl. Surf. Sci..

[18] Rui-Long, H. *et al.* Mechanism of cosmic ray high-energy particles charging test mass. *Acta Phys. Sin.* **70**. 10.7498/aps.70.20210747 (2021).

[19] Vocca H (2004). Simulation of the charging process of the LISA test masses due to solar flares. Class. Quantum Gravity.

[20] Armano M (2018). Precision charge control for isolated free-falling test masses: LISA pathfinder results. Phys. Rev. D.

[21] Inchauspé H (2020). Numerical modeling and experimental demonstration of pulsed charge control for the space inertial sensor used in LISA. Phys. Rev. D.

[22] Reames DV (1999). Particle acceleration at the sun and in the heliosphere. Space Sci. Rev..

[23] Tylka A (2005). Shock geometry, seed populations, and the origin of variable elemental composition at high energies in large gradual solar particle events. Astrophys. J..

[24] Ruan W-H, Liu C, Guo Z-K, Wu Y-L, Cai R-G (2020). The LISA-TAIJI network. Nat. Astron..

[25] Araújo H, Wass P, Shaul D, Rochester G, Sumner T (2005). Detailed calculation of test-mass charging in the LISA mission. Astropart. Phys..

[26] Vocca H (2005). Simulation of the charging process of the LISA test masses due to solar particles. Class. Quantum Gravity.

[27] Gong Y, Luo J, Wang B (2021). Concepts and status of Chinese space gravitational wave detection projects. Nat. Astron..

[28] Grimani C (2004). Cosmic-ray spectra near the LISA orbit. Class. Quantum Gravity.

[29] Heber B (1996). Spatial variation of $$>$$ 106 MeV proton fluxes observed during the Ulysses rapid latitude scan: Ulysses COSPIN/KET results. Geophys. Res. Lett..

[30] Smart D, Shea M (1999). Comment on the use of goes solar proton data and spectra in solar proton dose calculations. Radiat. Meas..

[31] NOAA National Centers for Environmental Information. GOES Space Environment Monitor Data. http://www.ngdc.noaa.gov/stp/satellite/satdataservices.html (2022).

[32] Rodriguez, J., Onsager, T. & Mazur, J. The east-west effect in solar proton flux measurements in geostationary orbit: A new goes capability. *Geophys. Res. Lett.* **37** (2010).

[33] Rodriguez J, Sandberg I, Mewaldt R, Daglis I, Jiggens P (2017). Validation of the effect of cross-calibrated goes solar proton effective energies on derived integral fluxes by comparison with stereo observations. Space Weather.

[34] Forbush SE (1946). Three unusual cosmic-ray increases possibly due to charged particles from the sun. Phys. Rev..

[35] Rodriguez J, Krosschell J, Green J (2014). Intercalibration of goes 8–15 solar proton detectors. Space Weather.

[36] Yang J, Zhang Z, Wei C, Lu F, Guo Q (2017). Introducing the new generation of Chinese geostationary weather satellites, FengYun-4. Bull. Am. Meteorol. Soc..

[37] Cane H, Lario D (2006). An introduction to CMEs and energetic particles. Space Sci. Rev..

[38] Laurenza M, Consolini G, Storini M, Damiani A (2015). The Weibull functional form for SEP event spectra. J. Phys. Conf. Ser..

[39] Reames D, Barbier L, Ng C (1996). The spatial distribution of particles accelerated by coronal mass ejection-driven shocks. Astrophys. J..

[40] Shannon CE (1948). A mathematical theory of communication. Bell Syst. Tech. J..

[41] Grimani C, Fabi M, Lobo A, Mateos I, Telloni D (2015). Lisa pathfinder test-mass charging during galactic cosmic-ray flux short-term variations. Class. Quantum Gravity.

[42] Armano M (2017). Charge-induced force noise on free-falling test masses: Results from LISA pathfinder. Phys. Rev. Lett..

[43] Vitale S (2002). Lisa and its in-flight test precursor SMART-2. Nucl. Phys. B Proc. Suppl..

[44] Luo Z, Guo Z, Jin G, Wu Y, Hu W (2020). A brief analysis to Taiji: Science and technology. Results Phys..

[45] Antonucci F (2012). Interaction between stray electrostatic fields and a charged free-falling test mass. Phys. Rev. Lett..

[46] Kenyon, S. P. *et al.* A charge management system for gravitational reference sensors–design and instrument testing. In *2021 IEEE Aerospace Conference (50100)*, 1–9 (IEEE, 2021).

[47] SILSO World Data Center. The International Sunspot Number. http://www.sidc.be/silso/ (2022).

[48] Birch MJ, Bromage BJI (2022). Sunspot numbers and proton events in solar cycles 19 to 24. J. Atmos. Solar-Terr. Phys..

[49] Nymmik R (2007). To the problem on the regularities of solar energetic particle events occurrence. Adv. Space Res..

[50] Nymmik R (2008). To the problem on the reliability of solar energetic proton flux models. Adv. Space Res..

[51] Singh, A. K. & Bhargawa, A. Predictions for solar cycles 25 and 26-a substantial weakening trend in solar activity. *43rd COSPAR Scientific Assembly. Held 28 January–4 February*, vol. 43, p. 1044 (2021).

